# A Bibliometric Analysis on Arrhythmia Detection and Classification from 2005 to 2022

**DOI:** 10.3390/diagnostics13101732

**Published:** 2023-05-13

**Authors:** Ummay Umama Gronthy, Uzzal Biswas, Salauddin Tapu, Md Abdus Samad, Abdullah-Al Nahid

**Affiliations:** 1 Electronics and Communication Engineering Discipline, Khulna University, Khulna 9208, Bangladesh; umamagronthy000@gmail.com (U.U.G.); salauddin.tapu@gmail.com (S.T.); nahid.ece.ku@gmail.com (A.-A.N.); 2 Department of Information and Communication Engineering, Yeungnam University, Gyeongsan-si 38541, Republic of Korea

**Keywords:** arrhythmia detection, bibliometric analysis, biblioshiny, PRISMA

## Abstract

Bibliometric analysis is a widely used technique for analyzing large quantities of academic literature and evaluating its impact in a particular academic field. In this paper bibliometric analysis has been used to analyze the academic research on arrhythmia detection and classification from 2005 to 2022. We have followed PRISMA 2020 framework to identify, filter and select the relevant papers. This study has used the Web of Science database to find related publications on arrhythmia detection and classification. “Arrhythmia detection”, “arrhythmia classification” and “arrhythmia detection and classification” are three keywords for gathering the relevant articles. 238 publications in total were selected for this research. In this study, two different bibliometric techniques, “performance analysis” and “science mapping”, were applied. Different bibliometric parameters such as publication analysis, trend analysis, citation analysis, and networking analysis have been used to evaluate the performance of these articles. According to this analysis, the three countries with the highest number of publications and citations are China, the USA, and India in terms of arrhythmia detection and classification. The three most significant researchers in this field are those named U. R. Acharya, S. Dogan, and P. Plawiak. Machine learning, ECG, and deep learning are the three most frequently used keywords. A further finding of the study indicates that the popular topics for arrhythmia identification are machine learning, ECG, and atrial fibrillation. This research provides insight into the origins, current status, and future direction of arrhythmia detection research.

## 1. Introduction

The heart is a vital organ of the human body that beats 2.5 billion times over a lifetime [1]. The heart usually beats about 75 beats per minute [2]. Various kinds of heart diseases cause interruptions to this normal heartbeat. Among various heart diseases, arrhythmia is one of the most critical conditions, which refers to any abnormal heart rhythm that cannot be physiologically justified [3]. The Sinoatrial (SA) node generates electrical impulses that regulate the heart’s rhythm. When these electrical signals are not functioning correctly, arrhythmia may occur. Such as malfunctioning can lead to various life-threatening conditions such as stroke, heart failure, and even death. Arrhythmia is one of the leading causes of death worldwide, which claims (15–20%) of all deaths [4].

There are several types of arrhythmias, such as ventricular fibrillation (VF), atrial fibrillation (AF), and ventricular tachycardia (VT). AF is the most ordinary form of arrhythmia. In 2010, approximately 33.5 million people were affected by AF worldwide [5]. According to the 2017 database, 3.046 million new cases of AF were recorded globally [6]. In high-income nations such as the United States, approximately 2–6 million people are impacted by AF, which is expected to double by 2060 [7]. According to recent statistics in the USA, AF causes more than 454,000 hospitalizations yearly [8]. AF is the reason for about 26,535 deaths per year in the USA [9]. People from underdeveloped and developing countries, such as Bangladesh, are affected more than developed countries, where 80% of all deaths occur due to this heart disease [10].

However, early detection and on-time arrhythmia prediction can reduce the risk of morbidity, hospitalization, and mortality. Due to the lengthy and expensive diagnosis process of the existing healthcare system, many state-of-the-art technologies are now being used to predict arrhythmias, such as statistical analysis, machine learning (ML), and deep learning (DL) based approaches. Research on arrhythmia detection has proliferated during the past few decades on a global scale. Due to this rapid growth, it is becoming more challenging for a researcher to stay consistent with all the most recent findings in arrhythmia detection and classification. Simultaneously, the most critical initiative is figuring out the future scope and the knowledge gap and knowing those factors which have received the most significant attention from researchers to detect and classify arrhythmia. Literature study is the most effective way to learn about these topics. The literature review provides an overview of existing research on a specific topic. We can do a literature review using several techniques, including a systematic review, meta-analysis, and bibliometric analysis.

We have chosen bibliometric analysis for the literature review in this study because it concisely provides much bibliometric information. It is a beneficial tool for literature reviews as it analyzes, tracks, and evaluates the quantitative connections and effects of publications in a particular field of study using mathematical and statistical techniques. This technique effectively identifies significant research, scholars, journals, institutions, and countries within a specific period and provides a quantitative overview of vast academic literature. Many researchers have already used bibliometric analysis for different biomedical fields, including hypertension [11], cancer [12], diabetes [13], heart disease [14], and stroke [15]. Even in arrhythmia, there have been several bibliometrics studies completed.

In 2022 S. Wang et al. [16] conducted a bibliometric analysis on ventricular arrhythmia (VA). They have studied 6897 VA-related papers, and the time duration of these papers was from 2001 to 2020. In 2020 S. Shi et al. [17] conducted a bibliometric analysis of atrial fibrillation (AF) using 21,839 research articles. They have found the USA the most productive country and Mayo Clinic the most influential institute. Authors in [16] have identified ventricular tachycardia, ventricular fibrillation, catheter ablation, implantable cardioverter defibrillator (ICD), and sudden cardiac death (SCD) as the most popular topics. J. Huang et al. [18] have studied the usage of AI for arrhythmia. They have used 636 papers that were published from 2004 to 2021. They have identified China as the most productive county and “deep learning”, “electrocardiogram (ECG)”, and” convolutional neural network” as the trending topics. Y. Ai et al. [19] have studied all the articles that analyze the relationship between depression and AF from 2001 to 2021.

S. Shi et al. [20] conducted a bibliometric analysis on cardio-oncology. For this purpose, they have used the articles from 2010 to 2022. From this analysis, they have found the USA as the most significant country based on publication, vascular disease, and atrial fibrillation are two popular topics for cardio-oncology. A. W. K. Yeung et al. [21] have used bibliometric analysis on the application of digital technology on cardi-ology. They mainly focused on relevant authors, affiliations, counties, journals, and trending topics. W. Shuaib et al. [22] conducted a bibliometric analysis based on the citation. According to their analysis, the USA is the most cited country, and “Circulation” is the most cited journal for cardiovascular articles.

Many authors have also studied bibliometric analysis of heart failure. Authors of the papers [23,24,25,26] have used different periods for their analysis, but they all have bibliometric analyses of heart failure. H. Wang [23] studied the period from 2009 to 2019. Shahid et al. [24] used the article published between 2001 and 2005. In [25], it is claimed that there exists a relationship between depression and heart failure. Authors of the paper [26] have used the articles from 1989 to 2021 for bibliometric analysis. They have all found the USA the most cited country for heart failure articles.

Though much bibliometric analysis has already been completed, we can see that no one has conducted bibliometric analysis on arrhythmia detection. In this paper, we have studied the bibliometric analysis of arrhythmia detection based on some significant factors, such as relevancy analysis, trend review, citation evaluation, and networking analysis. In addition, a wide range of indicators, such as bibliographic coupling, co-citation, co-occurrence of keywords, and collaboration, has been used for mapping the bibliographic data graphically.

This study is organized into five main sections. In Section 2, we have explained our proposed methodology for bibliometric analysis. Section 3 described leading authors, institutions, countries, the latest trends, and new arrhythmia detection and classification topics. Several types of network analysis are shown in the Section 4. In Section 5 and Section 6, we have summarized and concluded our work.

## 2. Methodology

**Figure 1:**
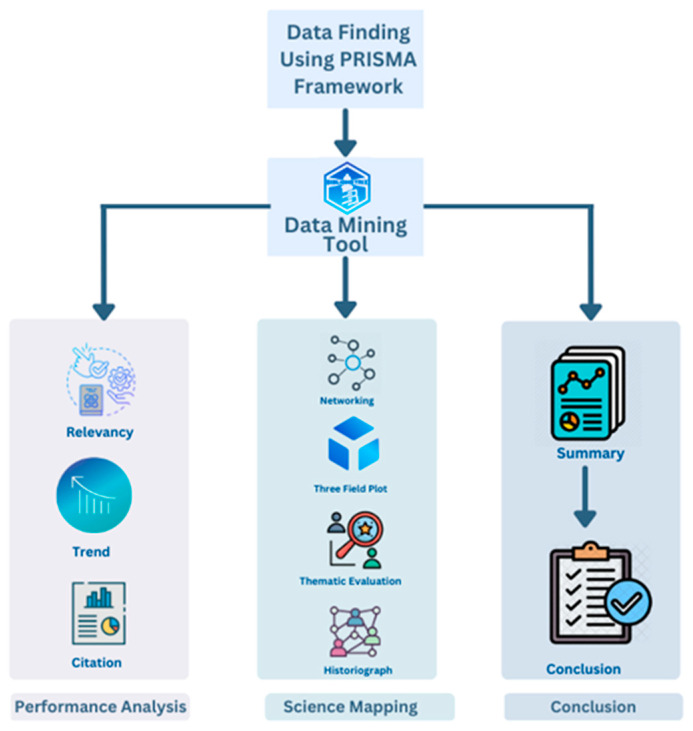
Proposed methodology for bibliometric analysis.

This paper performs a bibliometric analysis of arrhythmia detection and classification. Literature shows that this approach can give a broad overview of academic literature and can be effectively utilized to find influential research, writers, journals, groups, and countries across time. Nevertheless, because of the tremendous advancement in scientific technology, various bibliometric methods and applications are now developed to help researchers conduct their research. This rigorous approach to quantitative research analyzes the interconnections and the effects of publications in a particular field of research using statistical and mathematical methods. The term bibliometrics was first introduced by P. Otlet in 1934 and described as “the measurement of all aspects related to the publication and reading of books and documents” [27]. To perform bibliometric analysis, we have organized our work into three parts named data finding using PRISMA (Preferred Reporting Items for Systematic Reviews and Meta-Analyses) framework, data mining tool, and performance analysis. Figure 1 depicts our working steps.

### 2.1. Data Finding Using PRISMA Framework

For bibliometric analysis, we have selected the keywords “arrhythmia detection and classification” as our topic. Though much research has already been completed on this topic, we have selected this topic to investigate more about the historical development and current situation of arrhythmia detection and classification. This study will help the researchers find those authors, institutions, journals, and community members working in this research field with the highest contribution to arrhythmia detection and classification.

**Figure 2:**
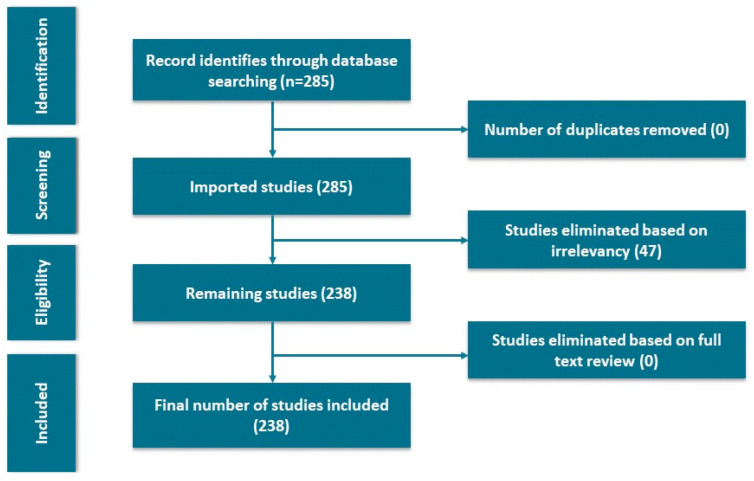
The PRISMA flow diagram.

The first step of our work is “data finding using the PRISMA framework”. PRISMA is a four-phase workflow representing the number of documents identified, selected, and discarded and the justifications for exclusions. The information flow across the various phases of a bibliometric review is described in PRISMA 2020 framework (Figure 2). Various search tools such as the Web of Science (WoS), Scopus, and PubMed are available to identify the relevant papers on a specific topic. As we do not have academic access to Scopus, we have used WoS as a search tool for this study. WoS is a research database that provides access to scientific and scholarly journals, articles, and conference papers. According to 2020, it was one of the largest databases in the world, with 74.8 million records [28]. For this analysis, we initially performed a search in the WoS database using the keywords “arrhythmia detection” and “arrhythmia classification” and the combined use of two keywords, such as “arrhythmia detection and classification”. The two key search parameters used in this process were the document type “article” and the research years “2005–2022”. In this step, we have identified 285 papers from the WoS database.

Though 285 papers on arrhythmia detection and classification have been published throughout this time, not all are related to our analysis. Subsequently, to make this analysis more appropriate, we have followed the principles of PRISMA proposed by Moher et al. [29]. Out of 285 papers, we did not have any duplicate papers. So, in the next screening stage, we imported 285 papers. The following step was eliminating irrelevant documents based on our research topic. An additional 47 articles were removed, reasoning their irrelevance, leaving 238 articles. As we did not have any articles based on the full-text review, 238 papers indicate the final selected papers for bibliometric analysis using the PRISMA method.

### 2.2. Data Mining Tool

The selected papers can be analyzed with the help of different data mining tools such as Biblioshiny, VOSviewer, Gephi, HistCite, and CiteSpace. For this study, we have selected the Biblioshiny software to analyze, evaluate, and develop graphical visualization from our database. Biblioshiny is an R statistical programming language tool developed by M. Aria and C. Cuccurullo [30] and designed for quantitative evaluation. The user-friendly interface of Biblioshiny makes it simple for users to import, modify, and generate interactive visualizations of data. A variety of visualization options are offered by Biblioshiny, such as bar graphs, line plots, and maps, which help researchers conduct their research. It can be integrated with other data mining software, including R and Excel, allowing users to evaluate further and manipulate data. We have performed all of our analyses through this software.

### 2.3. Performance Analysis

In order to evaluate the research performance, growth, and scientific trends in the field of arrhythmia detection and classification, we have used different bibliometric attributes such as citation analysis, trend analysis, network analysis, and others. Additionally, we performed this analysis from various bibliometric aspects, including the performance of the authors, journals, and institutions. In this paper, we have conducted two types of biblio-metric analysis. First, we did performance analysis, and next, we focused on science mapping. Performance analysis primarily considers the contributions of an individual, a group, or an organization to a particular research topic, and scientific mapping emphasizes visualizing the relationships and interconnections between different authors, journals, or institutes. Performance analysis is usually used to identify significant authors, sources, countries, or affiliations in a specific research field, and science mapping is used to figure out historical development, identify gaps in the literature, and identify emerging or declining research trends. In the following sections, we have explained performance analysis and science mapping on 238 selected papers.

## 3. Bibliometric Performance Analysis for Arrhythmia Detection and Classification

In bibliometric studies, the researcher’s contributions to a specific research field and the advancement of that field are investigated. A bibliometric performance analysis was conducted in this section based on various performance indicators, such as the number of publications, trends, number of citations, leading authors, institutions, and countries to provide a comprehensive knowledge and understanding of the myocardial arrhythmia detection and classification research from 2005 to 2022.

### 3.1. Leading Countries, Authors, Affiliations, and Sources Based on the Number of Publications

This section represents the most contributing countries, authors, institutions, and sources on myocardial arrhythmia detection and classification research based on the number of their publications.

#### 3.1.1. Most Productive Countries

**Figure 3:**
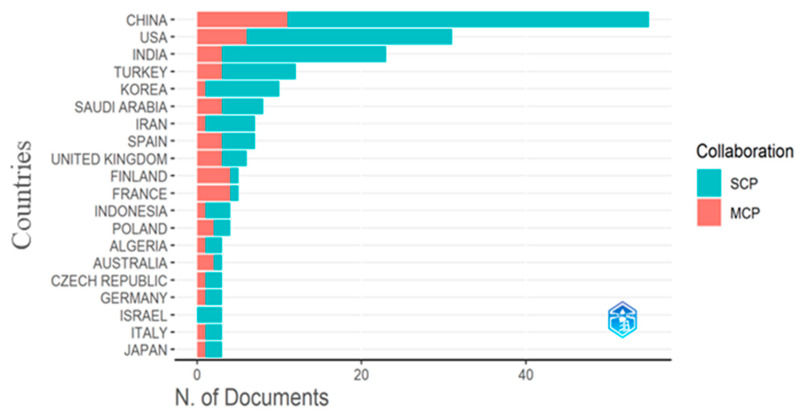
The most productive countries.

The nations that have published the most articles in this field are at the forefront of arrhythmia detection and categorization. Figure 3 represents the top 20 most productive countries or regions for arrhythmia detection and classification. In Figure 3, the blue box indicates single-country production, whereas the red box denotes multiple-country production from 2005 to 2022. The comprehensive documents include those from a single country and those created in association with other countries. China and the USA outperform all other countries based on their publication numbers, showing that they began their research efforts before most other countries worldwide. According to this analysis, China is the most productive country, and the USA is the second. China has 55 articles, whereas the total number of articles in the USA is 31, which is not close to the number in China. India is the third most productive country. It has 23 articles, almost two-thirds of the USA’s numbers. After India, other countries have a smaller number of articles on arrhythmia detection and classification. Korea and Turkey have published 12 articles and 10 articles, respectively. The bottom eight countries of Figure 3 have only three articles, significantly fewer than China. From this analysis, we can conclude that China has performed more research on arrhythmia detection and classification than any other country, and after China, the USA has shown more interest in that area.

#### 3.1.2. Most Relevant Authors

**Figure 4:**
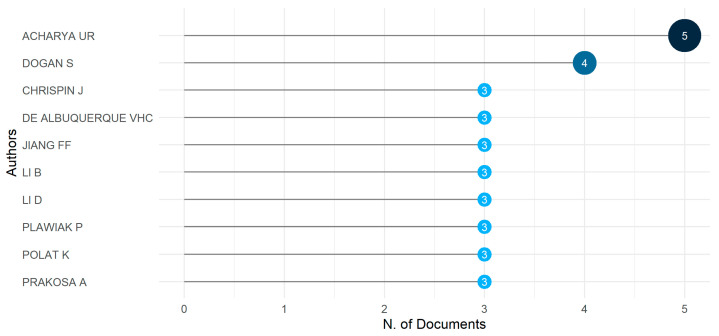
The most relevant authors.

In academic research or writing, a relevant author’s work relates to the discussed topic and can provide valuable insights or information. Several authors around the world have conducted research in the field of arrhythmia. Regarding the analysis at the author’s level, the most relevant author is one of them. Figure 4 lists the top 10 most relevant authors based on their contributions to arrhythmia research in terms of published articles. The author named U. R. Acharya [31,32,33,34,35], ranks first with five contributions, and S. Dogan [35,36,37,38], contributes four publications. The remaining eight authors contributed equally with three publications. From this analysis, we can say that all authors’ contributions in the research field of arrhythmia are closely related.

#### 3.1.3. Most Relevant Affiliations

**Figure 5:**
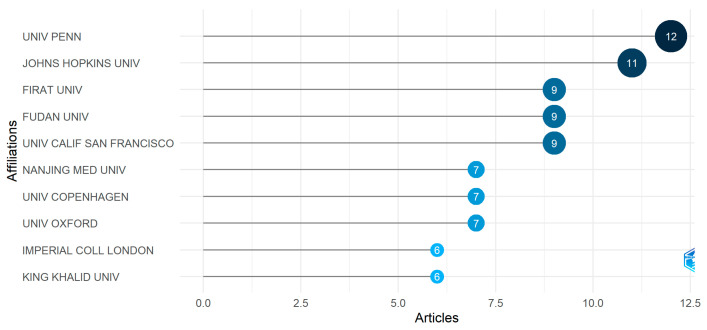
The most relevant affiliations.

Further, the most relevant affiliations were also investigated. A relevant affiliation is an organization or institution related to where similar arrhythmia research is being worked on. We classify the top affiliations based on the number of research and published articles on arrhythmia. In Figure 3, it has been shown that the USA is one of the most productive countries based on the number of published papers. Again, the analysis of the most relevant affiliations (Figure 5) reveals that the University of Pennsylvania, located in the USA, is the most relevant affiliation with 12 published articles. The Johns Hopkins University in the USA is the second most relevant institution with 11 publications. Firat University in Turkey, Fudan University in Shanghai, China, and the University of California in the USA were three productive institutions, with nine publications each. The rest of the top-listed universities have fewer publications than those mentioned above. Of the top 10 universities, four are from the USA and China.

#### 3.1.4. Most Relevant Sources

**Figure 6:**
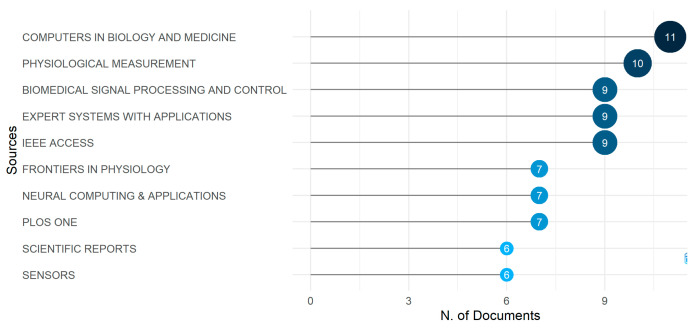
The most relevant sources.

Analyzing the most relevant sources is an essential tool for evaluating the performance of a journal in a specific field. Figure 6 lists the name of the ten journals that have published the most papers on arrhythmia research. The journal “Computer in Biology and Medicine” has achieved the top position with 11 publications. The second prolific source is the “Physiological Measurement” journal which focuses on clinical research and practice. This source has published ten papers on arrhythmia detection and classification. The following three journals, named “Biomedical Signal Processing and Control”, “Expert System with Applications”, and “IEEE Access”, have achieved equal positions by publishing nine papers.

This study reveals all the journals that publish arrhythmia-related papers. Interestingly, there is hardly any difference in performance among these top five journals. Compared to the leading five journals, the rest of the journals named “Frontiers in Psychology”, “Neural Computing and Applications”, “PLOS ONE”, and “Scientific Reports and Sensors” have fewer publications. The last two journals in Figure 6 have published six papers each.

### 3.2. Trend Analysis

Trend analysis in academic research involves identifying and analyzing patterns or trends in a specific research area over time. It can be helpful for various purposes in academic research, such as determining whether certain factors or variables are changing over time that provides insight into the relationships between different variables in the data.

#### 3.2.1. Word Cloud of Keywords

**Figure 7:**
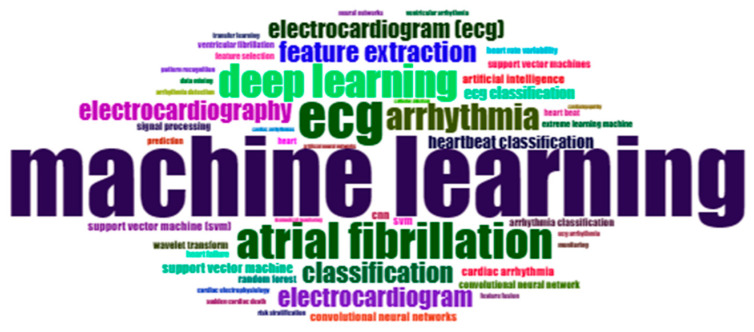
Word cloud of keywords.

Figure 7 represents the 50 most frequently used author keywords using a word cloud where the size of each keyword represents its frequency. The term “machine learning” has been used most frequently in arrhythmia research from 2005 to 2021. Most researchers preferred the machine learning approach during that time for their arrhythmia research. “ECG” is the second most explored keyword after machine learning. The term “Deep learning”, “atrial fibrillation”, and “arrhythmias” all have smaller physical dimensions than “ECG”, indicating that these three terms are used less frequently than the term “ECG”. In addition, the other available keywords in the word cloud are not used very often; their occurrence rate is insignificant.

#### 3.2.2. Growth of Top 10 Author’s Keywords

**Figure 8:**
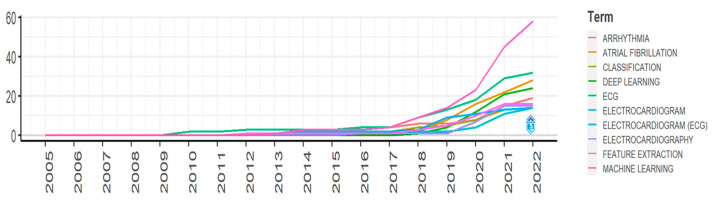
Growth of top 10 keywords for arrhythmia detection.

Figure 8 depicts the growth of the top 10 author’s keywords from 2005 to 2022, and the keywords are “arrhythmia”, “atrial fibrillation”, “classification”, “deep learning”, “ECG”, “electrocardiogram”, “electrocardiogram (ECG)”, “electrocardiography”, “feature extraction”, and “machine learning”. During that time, every keyword has experienced a significant gradual increase in growth rate. The keyword has an annual growth, and researchers must be aware of the most recent development. In this figure, X-axis represents the year, and the Y-axis represents the number of cumulative occurrences. The keyword “machine learning” has an excellent performance curve in the arrhythmia detection and classification research field. This keyword has the highest growth rate in this field. Though the term “machine learning” started its journey in 2005, its growth rate reached 58 by the end of 2022. None of the keywords, except “machine learning”, we chose to analyze had ever been used from 2005 to 2009. Further, in 2010, the term “ECG” first came to the author’s attention, which is currently in the growth list and the second position. Its cumulative occurrences are 32, which is exceptionally low compared to the keyword “machine learning”. In 2022, the third highest growth keyword was “atrial fibrillation”, used 28 times. “Deep learning” has the fourth highest growth rate and is close to “atrial fibrillation”. After “deep learning”, arrhythmia obtains the next growth rate. It is noteworthy that “feature extraction”, “classification”, “electrocardiography”, “electrocardiogram”, and “electrocardiogram (ECG)” have almost equal growth rates.

#### 3.2.3. Trending Topics

**Figure 9:**
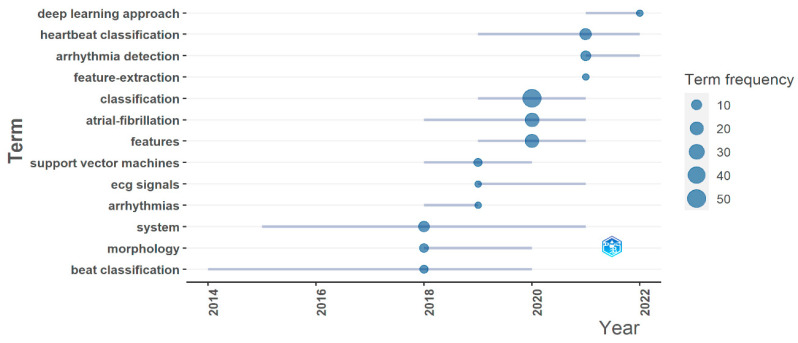
Trending topics for arrhythmia detection.

Generally, trending topics in research are changed every year, which determines the research interest. Figure 9 shows the trending topics from 2014 to 2022 in arrhythmia detection and classification. As indicated in Figure 9, the size of the blue circle indicates a topic’s frequency. It starts from 10, represented by the smallest circle, and the highest frequency is 50, denoted by the largest circle. At the beginning of the arrhythmia research, the trending topic was “beat classification”, and it predominated from 2014 to 2020. In 2016, the “support vector machines” emerged, and its trending duration lasted till 2020. In 2018, “beat classification”, “morphology”, and “system” got researchers’ attention. However, among these three topics, “system” was able to attain the most significant attention. It is interesting that in 2019, three topics, “ECG signals”, “support vector machine”, and “arrhythmias”, had the same popularity. In the following year, the most important topic was “classification”, which beats the two crucial topics “atrial fibrillation” and “feature”. Again in 2021, three topics equally attract scholars’ attention. These three topics were “neural network”, “heartbeat classification”, and “arrhythmia detection”. “Deep learning approach” is the only hot topic of 2020, but its popularity is extremely poor. This analysis shows that the topic “beat classification” was on trend for the most prolonged period. However, we hope that “arrhythmia detection” and “deep learning approach” will be on trend in the future because, among all the topics, these two topics have only been visible since 2021.

### 3.3. Citation Analysis

Citation analysis is an essential factor in the field of bibliometrics. It studies the effect and significance of research and scholarly output. It is performed by looking at how frequently a work is cited, where and by whom it is cited, and the context in which it is cited. This analysis can be used for investigating the influence of a particular author and work. Additionally, it evaluates the quality and determines the importance and relevance of research work to a particular topic.

#### 3.3.1. Most Cited Countries

**Figure 10:**
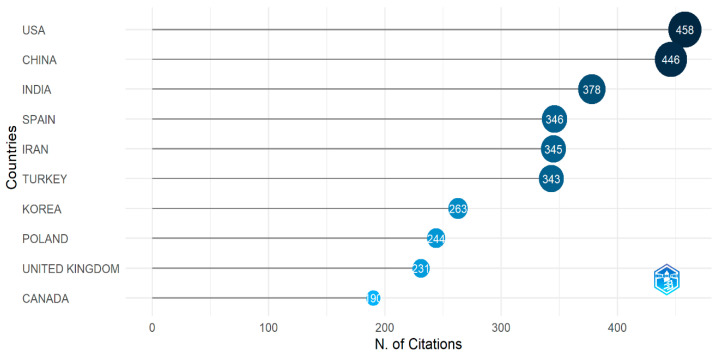
The most cited countries.

Citation analysis is a highly effective tool for determining the research significance of an article. Figure 10 represents the top 10 most cited countries in the world. Although China published the highest number of papers on arrhythmia detection and classification, the papers published in the USA are the most cited. The reason is that the paper published in the USA is more relevant to arrhythmia classification and detection and contains more valuable information. When ranked by the number of citations, the USA has the most significant scholarly impact with 458 citations, followed by China with 446 citations. India is the third most cited country in the world for arrhythmia research. We have found 378 papers published on arrhythmia detection and classification from India. The exciting thing is that middle eastern countries, such as Iran and Turkey, are ranked in the middle of the list. They have around 350 citations each. Canada ranks last among the ten countries with 190 citations. It is noteworthy to mention that most articles on the detection and classification of arrhythmia have come from the USA and China.

#### 3.3.2. Most Cited Authors

**Figure 11:**
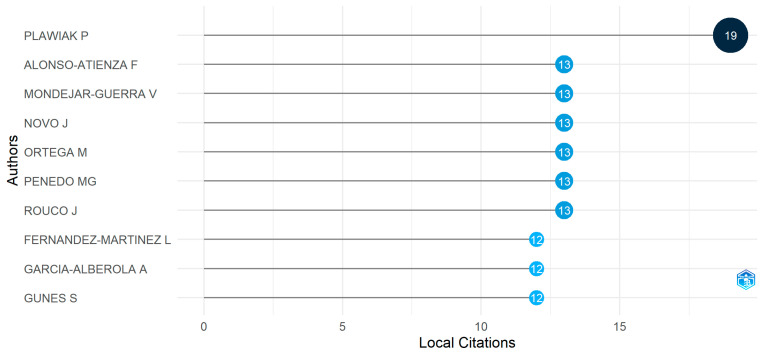
The most cited author.

The following exciting analysis is the most cited author, which carries considerable significance in evaluating the performance of an author in a specific research field. Figure 11 displays the name of the 10 most locally cited authors in the field of arrhythmia classification and detection with their citation numbers. The author named P. Plawiak [32] ranks first and received 19 citations, the highest number of citations during 2005—2022. It is exciting that after P. Plawiak, six authors achieved the second position. They all have 13 citations each. The names of these six authors are F. Alonso-Atienza [39], ‪V. M. Mondéjar-Guerra [40], J. Novo [40], M. Ortega [40], ‪M. Gonzalez Penedo [40], and ‪J. Rouco [40]. Lastly, we can see that three of the remaining authors have been cited 12 times in several publications. From this figure, we can conclude that each author has at least 12 publications but not more than 20.

#### 3.3.3. Most Cited Sources

**Figure 12:**
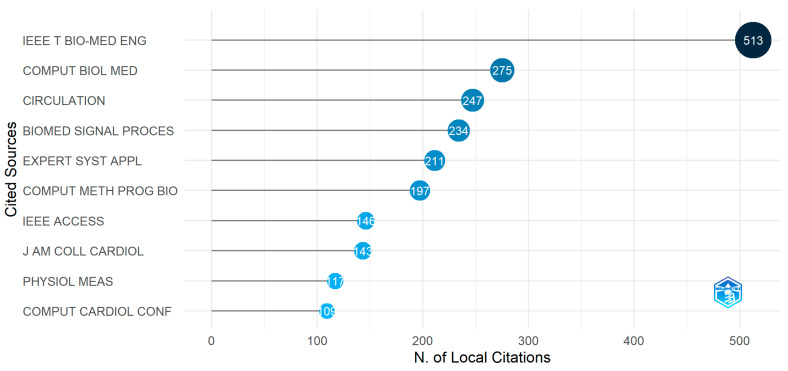
Most cited source.

Analyzing most cited sources plays an essential role in citation analysis. The most cited sources refer to those cited most frequently by other authors in their research. We can understand a journal’s impact on specific research work by analyzing the most cited sources. Figure 12 shows that the journal “IEEE Transaction on Biomedical Engineering” has the most citations, 513 times. Much research has referred to this journal’s publications, making it a significant arrhythmia detection and classification journal. Another major journal is “Computers in Biology and Medicine”. Around 275 research papers on arrhythmia have cited the publications of this journal. The third most cited journal, “Circulation”, has 247 citations. Besides the three most cited journals, there are “Biomedical Signal Processing and Control”, “Expert Systems with Applications”, and “Computer Methods and Programs in Biomedicine”, with citations of 234, 211, and 197 each. Though the number of citations in these journals is not high as “IEEE Transaction on Biomedical Engineering”, the papers of these journals also have a decent number of citations, which makes them one of many major publications. A few noteworthy journals are “IEEE Access”, “Journal of the American College of Cardiology”, and “Physiological Measurement and Computing in Cardiology” with citations of 146, 143, 117, and 109, respectively. Analyzing this figure, we can conclude that most authors have cited the journal “IEEE Transaction on Biomedical Engineering”.

## 4. Science Mapping

Science mapping is an approach for identifying and visualizing the connections between various scientific concepts and research domains. Usually, science mapping represents a network diagram. These network diagrams and their connections can be based on various analyses, including the patterns of citations between papers, the collaboration of institutes in research publications, or the co-citation of authors.

### 4.1. Networking Analysis

Network analysis is a method of analyzing the relationships between various journals, affiliations, and countries. In academic research, network analysis can be conducted in several ways, such as through co-citation networks of journals and collaboration networks of institutes. One of the main advantages of network analysis is that it helps researchers identify chances for collaborative learning or teamwork with other researchers working on related subjects.

### 4.2. Collaboration Network of Authors

**Figure 13:**
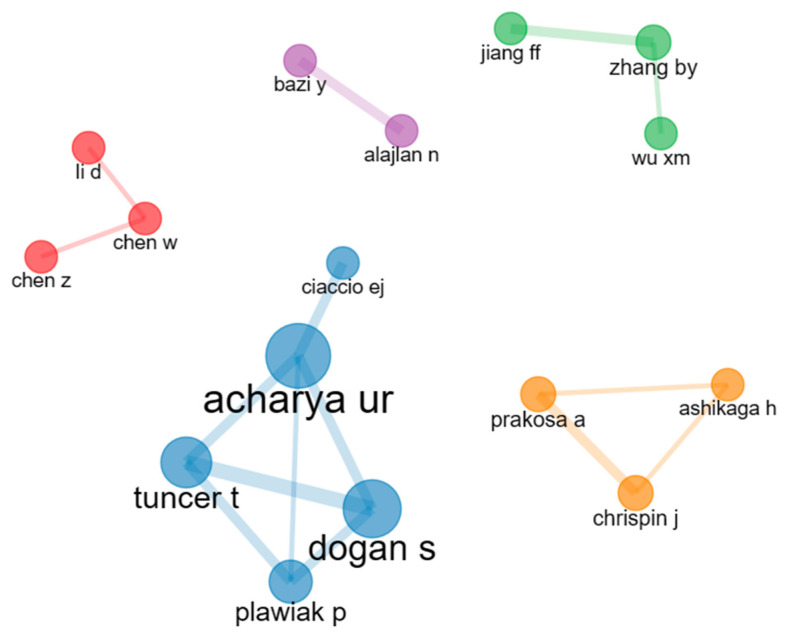
Collaboration network of authors.

A collaboration network of authors indicates the collaborative association between different authors within a specific field of research. Figure 13 shows the collaboration network of authors for arrhythmia detection. In this figure, different colored clusters represent the different collaborative groups, and the node size represents their contribution to this research topic. There are five collaborative groups (blue, red, green, orange, and purple). From this figure, we can see that the blue-colored group is the largest collaborative group. In this group, five different authors are working together. Among them, U. R. Acharya [31], S. Dogan [37], and P. Pławiak [41] have contributed more than E.J. Caiccio [42]. The orange, green, and red clusters consist of three authors. In the orange cluster, each author collaborates with the other two authors. Authors named A. Prakosa [43], J. Chrispin [43], and H. Ashikaga [44] have worked together to predict arrhythmia. The remaining clusters are similarly formed. The purple cluster is the smallest collaborative group of this figure. Only two authors, Y. Bazi [45], exist in this group, and N. Alajlan [45]. Analyzing this figure, we can conclude that the authors of the blue cluster have created a strong collaboration network between them, and U. R. Acharya [31] has the highest contribution to his group.

### 4.3. Collaboration Network of Countries

**Figure 14:**
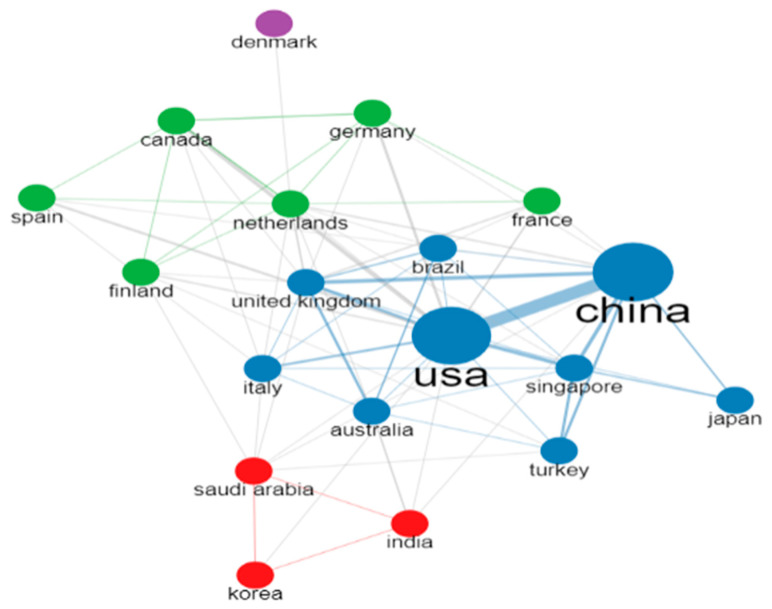
Collaboration network of countries.

Figure 14 shows the collaboration network between the countries that have worked on arrhythmia detection and classification. In this figure, different colored (red, blue, green, and purple) circles and lines are used to identify the collaboration among the countries. The connection between countries of the same color indicates their collaboration and the line’s thickness represents the strength of collaboration. At the same time, the size of the circle also indicates their collaboration frequency. According to this figure, China and the USA have more collaborative partners than any other country, and their collaboration is strongest. In addition, the blue cluster is the largest cluster among the other clusters, indicating that more countries are cooperating to detect and classify arrhythmia under this cluster. There are six countries under the green cluster, meaning Canada, Spain, Finland, Germany, Netherlands, and France are working together. The red cluster contains three countries, and the purple cluster has only Denmark, which collaborates with only the Netherlands. Therefore, it can be concluded that the most significant networks are in the blue cluster.

### 4.4. Collaboration Network of Institutions

**Figure 15:**
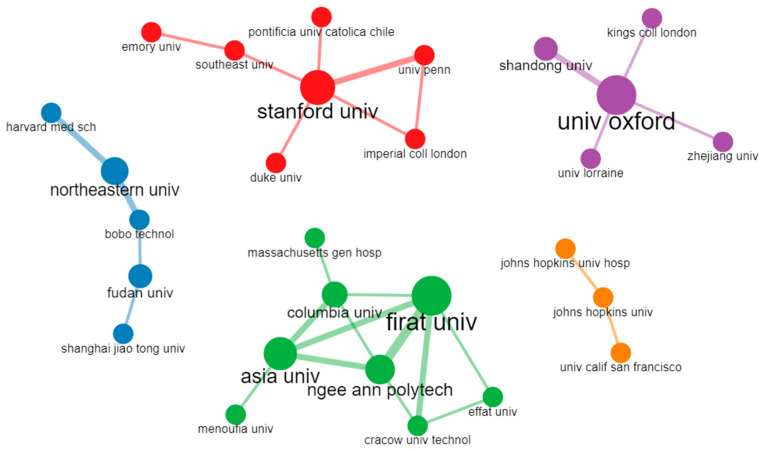
Collaboration network of institutions.

The collaboration network of the institutions in the research field of arrhythmia detection and classification is shown in Figure 15. In this figure, the institutions are represented by colored circles. The circle’s size indicates the publication frequency from a specific institution. Lines of the same color represent the collaboration between institutions. Based on their collaborative research activities, there are five clusters with five colors: red, blue, green, orange, and purple. Every institution in a specific cluster has conducted its research activities in collaboration with other institutions, and they may be in the same or different geographical areas. In the figure, the green cluster is the largest. In this cluster, eight universities are from different geographical areas, such as Firat University from Turkey, Columbia University from the USA, and Effat University from Saudi Arabia, and they have worked in close collaboration with each other. Firat University has developed the highest collaborative effort with other universities. In the red cluster, Stanford University, Duke University, University of Pennsylvania, and Emory University are in the USA, Imperial College London is in the UK, Pontifical Catholic University is in Chile, and Southeast University is in China. The rest of the clusters are also developed in the same way. Therefore, from this figure, we can say that the institutions of green clusters have a strong collaboration, and Firat University from Turkey is the most collaborative institution.

### 4.5. Co-Citation Network of Journals

**Figure 16:**
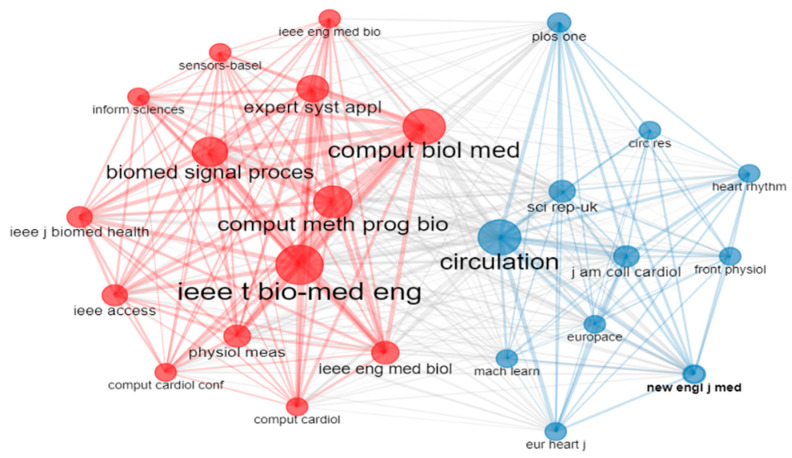
Co-citation network of journals.

Now, to understand the relationships between different journals, we will explore the co-citation network of journals. When two publications from separate journals are cited in the same article, this is known as a co-citation of journals. Figure 16 shows the co-citation network of the journal, and it contains 25 journals. Figure 16 has two clusters, which are made of two distinct colors, one cluster is red, and another is blue. The red cluster may represent journals focusing on biology with technology, while another cluster may represent journals focusing on machine learning-based cardiovascular disease prediction. The colored nodes represent the journals, and the line between them indicates the co-citation between the two journals. The size of the nodes represents their citation frequency. From Figure 14, we can see that the red clustered journals “IEEE Transaction on Biomedical Engineering (IEEE t bio-med eng.)”, “Computers in Biology and Medicine (comput biol med)”, and the blue clustered journal “Circulation” are the most cited, and their network of citations is vast. Some journals, such as “Computer Methods and Programs in Biomedicine (comput meth prog bio)”, “IEEE Transaction on Biomedical Engineering (IEEE t bio-med eng.)”, “Computers in Biology and Medicine (comput biol med)” and so on have cited each other papers and made the red cluster. The blue cluster also develops according to their citation and linkage with one another. In the case of arrhythmia detection and classification, researchers can track changes in the relationships between different journals by creating co-citation networks and finding the most influential journals in this research field. Analyzing this figure, we can conclude that there exists a strong relationship between the journals named “IEEE Transaction on Biomedical Engineering (IEEE t bio-med eng.)”, “Computers in Biology and Medicine (comput biol med)”, “Computer Methods and Programs in Biomedicine (comput meth prog bio)”, and “Biomedical Signal Processing and Control”.

## 5. Summary of Performance Analysis

The complete overview of different performance analyses is given in this section using a thematic evaluation, three-field plot, and historiography. It also shows the relationship between different analyses.

### 5.1. Thematic Evolution

**Figure 17:**
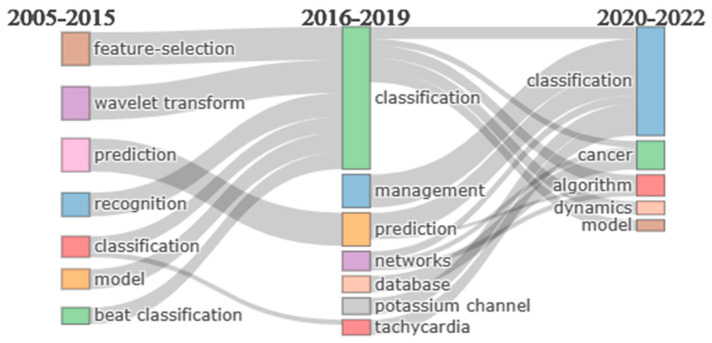
Thematic evolution for arrhythmia detection.

The analysis of thematic evolution is another imperative topic, shown in Figure 17 using a Sankey diagram. A Sankey diagram is a graphical representation of a flow of information, resources, and other quantities between nodes in a system [46]. The width of the lines that connect the nodes in a Sankey diagram is proportional to the amount of flow [46]. Each box, called a node, denotes one theme in this figure. The height of the box denotes the occurrence rate of each theme. This diagram represents the connection between different themes.

Notably, there is a variation between themes as the scholars preferred different themes at various times. We can see that though the “classification” emerged between 2005 and 2015, researchers have become more interested in this theme from 2016 to 2019. Furthermore, from 2020 to 2022, it could keep the researchers’ interest almost the same way. Initially, some themes were quite popular such as “feature selection”, “wavelet transform”, “recognition”, “model”, and “beat classification”. However, over time, each of these melded into “classification”, which attracted additional researchers to the “classification”. Some themes that appeared from 2016 to 2019, such as “management”, “network”, “database”, and “tachycardia”, did not develop later as a unique theme but instead combined with “classification”. Analyzing this figure, we can see that from 2005 to 2022, the only theme that remained consistent was classification, and it had the highest occurrence frequency from 2015 to 2019.

### 5.2. Three Fields Plot

**Figure 18:**
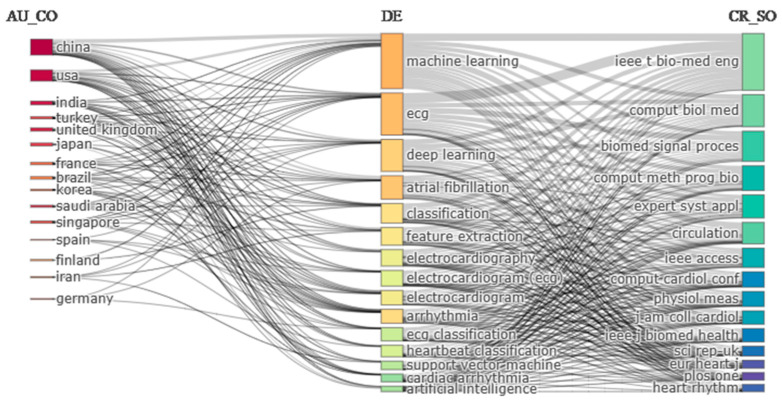
Three fields plot for arrhythmia detection.

The three fields plot included in the Bibliometrix tool enables comprehension of the entire bibliometric analysis in a single figure. It represents a significant relationship between the different analysis subjects for arrhythmia detection and classification. Figure 18 shows the proportionality among the 15 most active countries, frequently used keywords of those countries and primary journal sources for detecting and classifying arrhythmia. The left field of Figure 18 represents the 15 most active countries, the center field shows the 15 keywords, which get the maximum attention of the researchers of those countries, and the leading journal sources are included in the right field.

Figure 18 also represents a meaningful correlation between the number of publications in the country, the most widely used keywords, and the leading journal source. Most arrhythmia detection and classification papers are published in China and the USA. After these two countries, India and Turkey have published many research articles, respectively, shown in Figure 3. However, from this figure, we can say that “machine learning” seems to be the essential keyword for researchers. “ECG”, “deep learning”, “atrial fibrillation”, and “classification” are also quite popular among researchers, as represented in Figure 8.

From the right field, we can see that “IEEE Transaction on Biomedical Engineering (IEEE t bio-med eng.)”, “Computers in Biology and Medicine (comput biol med)” and “Biomedical Signal Processing and Control (biomed signal process)” are the primary journal sources. They have published many articles on arrhythmia detection and classification.

### 5.3. Historical Direct Citation Network

**Figure 19:**
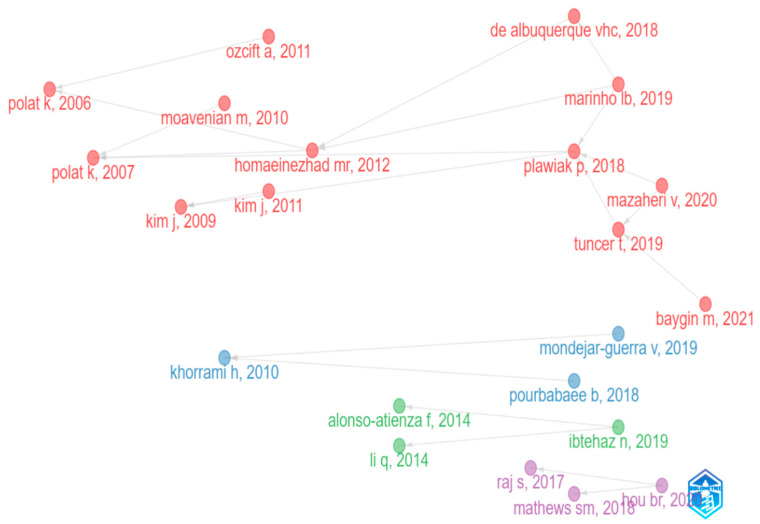
Historical direct citation network.

Figure 19 displays the bibliometric historiography for arrhythmia detection and classification. A historical citation network is a graphical representation that visualizes the relationships between authors based on how others cite them. Each color stream denotes the direct citation with their historical development. They highlight the significance of a particular keynote. The size of the stream depends on the number of cited documents in the same concept. In this network, the nodes represent the individual author’s name with the publication year, and the edges between the nodes represent the citations that one author makes to another. The length of the edge between two nodes denotes the period. According to Figure 9, the red stream is the most significant citation stream, which started with the research of K. J. E. S. A. Polat in 2006 [47] and ended with the research of M. Baygin in 2021 [37]. This stream focuses on arrhythmia classification. The most cited author P. Pławiak [32], who has 12 citations, is from the red steam. He proposed a novel methodology named “1 type of normalization x 2 Hamming window widths x 4 types of classifiers in the field of arrhythmia classification in 2018. The remaining three streams have three nodes each. However, among them, the blue stream is bigger than the other two. H. Khorrami [48], who has contributed to the identification of ECG arrhythmias using discrete wavelet transform (DWT), continuous wavelet transforms (CWT), and discrete cosine transform (DCT) transformations, and ended with V. M. Guerra’s [40] study on heartbeat categorization. The smallest stream is the purple stream. The period of this stream was from 2017 to 2020. This stream is based on ECG analysis for arrhythmia classification. S. Raj [31] has worked on ECG signal analysis through PSO (Particle Swarm Optimization) optimized SVM (Support Vector Machines) and DCT-based DOST (Discrete Orthogonal Stockwell Transform). In 2018, S. M. Mathews [49] worked on ECG classification. Both of these publications were referenced by the author B. Hou [50] in his paper in 2020. According to this graph, some researchers are working on detecting arrhythmia, while others are working on classifying arrhythmia for a particular period and mentioning the relevant papers they used for their research.

## 6. Conclusions

Arrhythmia is a severe condition that can result in several complications, including stroke, heart failure, and even death. Accurate detection and classification of arrhythmia can help determine an individual’s prognosis, which can help reduce the risk of complications and improve outcomes. Much research has been completed so far to predict and classify arrhythmia accurately. This paper describes the bibliometric analysis of arrhythmia detection and classification. Two different bibliometric methods, named “performance analysis” and “science mapping”, have been used in this paper.

Publication analysis, trend analysis, and citation analysis have received the highest priority in this research for performance analysis. By analyzing 238 papers on arrhythmia detection and classification from 2005 to 2022, we can see that China’s contribution beats the USA, India, and all other countries in this research field. The author, named U. R. Acharya from India, has been the most relevant author, and P. Plawiak is the most cited author over the past 17 years. Regarding the contribution of an individual institution, the University of Pennsylvania in the USA has been completed and published the maximum number of arrhythmias detections and classification-related research. For detecting and predicting arrhythmias, “machine learning” has been the most used keyword in this technological era for the past 17 years. However, the other popular keywords are “ECG”, “atrial fibrillation”, and “deep learning”. According to recent statistics, “deep learning approach”, “heartbeat classification”, and “arrhythmia detection” are now in trend, and researchers are paying more attention to those topics.

Later, we used science mapping to visualize and analyze the relationships and connections more thoroughly between different authors, countries, institutions, and journals. In order to explain science mapping, we have focused more on the co-citation network of journals, collaboration network of institutions, collaboration network of countries, and collaboration map for different continents and subcontinents. By creating a collaboration network for 20 countries, we can see that there are four unequal groups, and each group member is working in collaboration to achieve accurate results in arrhythmia detection and classification. China and the USA have developed the best collaboration network among themselves. Similarly, 28 institutions from different countries continue their research by organizing themselves into five clusters. The Turkish university, Firat University, has established collaborative relationships with five other institutions, which makes it the most cooperative institution. The journals “IEEE Transaction on Biomedical Engineering”, “Computers in Biology and Medicine”, “Computer Methods and Programs in Biomedicine”, and “Biomedical Signal Processing and Control” have a significantly close connection among themselves. We have taken the help of thematic evaluation, a three-field plot, and a historical direct citation network to provide a comprehensive overview of arrhythmia detection and classification. From 2005 to 2015, topics such as feature selection, wavelet transform, and prediction got the highest priority in detecting and classifying arrhythmia. However, in the following three years, “classification” became the most popular topic, followed by “management” and “prediction”, and “classification” has managed to retain its popularity in the last three years as well. This outcome gives us information on the emerging trends in detecting and predicting arrhythmias. The three fields plot, and historical direct citation network summarize previous analyses.

This study gives the researchers, authors, and others a complete overview of various aspects of research for arrhythmia detection and classification using a bibliometric method. This paper recognizes the significant authors, institutions, countries, and journals working day and night for better accuracy and improving arrhythmia detection and classification. Furthermore, this study has highlighted the latest trends and past status, making research easier for new investigations.

## Data Availability

The research data is available on special request to the first author of this article.
